# Dietary nanocomposite of vitamin C and vitamin E enhanced the performance of *Nile tilapia*

**DOI:** 10.1038/s41598-024-65507-1

**Published:** 2024-07-08

**Authors:** Ahmed H. Sherif, Riad H. Khalil, Talaat S. Talaat, Mohamed Z. Baromh, Mahmoud A. Elnagar

**Affiliations:** 1 Fish Diseases Department, Animal Health, Research Institute AHRI, Agriculture, Research Centre ARC, Kafrelsheikh, Egypt; 2 Fish Diseases Department, Faculty of Veterinary Medicine, Alexandria University, Alexandria, Egypt; 3 Division of Aquaculture, National Institute of Oceanography and Fisheries (NIOF), Alexandria, Egypt

**Keywords:** Chitosan, Nanoparticles, Vitamin C, Vitamin E, *Oreochromis niloticus*, Growth, Innate immunity, Antioxidant, Drug discovery, Immunology

## Abstract

Nowadays, nanomaterials enter high numbers of daily used products and drug manufacture. A nanocomposite of vitamins C (VC) and vitamin E (VE) with chitosan as a vehicle and protector was used in a comparative eight-week feeding study, Nile tilapia weighing 31.2 ± 0.36 g distributed in seven groups and fed (G1) basal diet, (G2) bulk VC, (G3) VC- nanoparticles (NPs), (G4) bulk VE, (G5) VE-NPs, bulk VCE (G6), and (G7) VC plus VE (VCE)-NPs, respectively. The Nile tilapia-fed nanocomposite vitamins had significantly higher growth performance compared to the control; VCE-NPs had the superiority among tested supplementations where total weight gain (63.6 g), daily weight gain (1.13 g), relative growth rate (206.1%) with lower feed conversion rate (1.6) and insignificant feed intake (101.5 g). Overall, the level of liver enzymes was significantly decreased in fish serum after eight-week nanocomposite supplementation, and dietary VCE-NPs caused a significant reduction of serum AST (18.45 IU/L) and ALT (14.77 IU/L) compared to the control 25.5 IU/L and 17.6 IU/L, respectively. Fish fed dietary VCE-NPs, VC-NPs, and VE-NPs had significant enhancement of RBCs 4.2 × 10^6^/μL, 3.8 × 10^6^/μL, and 3.55 × 10^6^/μL; WBCs 46.15 × 10^3^, 42.9 × 10^3^, and 44 × 10^3^/μL, respectively, Also TP was significantly higher 6.38 g/dL in VCE-NPs group compared to the control and the other treatments. Over all, the dietary nanocomposite vitamins boost the innate immunity of the experimental Nile tilapia, the oxidative burst activity (OBA), phagocytic activity (PA), phagocytic index (PI), and serum antibacterial (SAA) were significantly increased compared to those received bulk vitamins and the control. The activity of antioxidant biomarkers in fish serum including glutathione peroxidase (GPx), catalase (CAT), superoxide dismutase (SOD), total antioxidant capacity (TAC), glutathione reductase (GR), and myeloperoxidase (MPO) showed a rise in the serum of Nile tilapia received nano- and bulk-form of VC and VCE compared to the control and both forms of VE. Furthermore, the level of malondialdehyde (MDA), reduced glutathione (GSH), and oxidized glutathione (GSSG) were significantly increased in the fish serum following the trend of antioxidants enzymes. In conclusion, a dietary nanocomposite of vitamin C and vitamin E enhanced Nile tilapia's growth performance and feed utilization. It could also improve health status and immune response. The values of antioxidant biomarkers indicated that the nanocomposite could help the fish body scavenge the generated reactive oxidative species (ROS).

## Introduction

Tilapia species originated in Africa (their native homeland) after they were globally distributed in the 1900s^1^. Tilapia is widely recognized as one of the favorite fishes to be farmed worldwide, it is classified as the second most widespread species whose production is increasing every year, as it requires low maintenance cost, withstands adverse environments, is disease-resistant, and is fast-growing to marketable size ^1,2^. Feed cost, quality, and quantity remain the biggest challenges facing the aquaculture industry in Egypt, one of the largest producers of fish, the number one producer in Africa, and the third producer of Nile tilapia worldwide^3^.

Vitamins are organic compounds essential for fish’s health, growth, and survival. Vitamin E (VE) is an organic substance that fish bodies cannot synthesize, so it needs to be added to the fish diet. It is a fat-soluble material with antioxidant properties protecting body cells from generated reactive oxygen species (ROS). Also, alpha-tocopherol is one of eight different forms of VE, possessing significant impacts on fish health; it is considered one of the essential nutrients affecting fish immunity, where a dietary supplement with VE efficiently can decrease mortality as well as increase fish growth rate and fertility^4^. Also, VE improves both specific and nonspecific immunity. Besides, it has an essential role in relieving oxidative stress in fish due to being a powerful antioxidant that protects from oxidative injury to various fish tissues ^5^. Furthermore, it could preserve the leukocyte functions^6^.

Vitamin C (VC) is not biosynthesized in fish, so it needs to be added to the diets of farmed fish, it is also thermolabile and rapidly degraded in water^2,4^. VC is a well-known antioxidant that significantly influences the immune system, modulating and enhancing immune responses^7^ by improving the proliferation of B and T lymphocytes, decreasing histamine, and stimulating cytokine synthesis^8^.

Fish need VC for normal growth behavior but cannot biosynthesize VC.

Chitosan is a natural polymer obtained from the crustacean exoskeleton. It has advantageous properties such as being biodegradable and biocompatible; therefore, it is considered an attractive candidate as a vehicle used in medicines delivery inside the animal body, as it could encapsulate vaccine molecules. Interestingly, it could act as an immune stimulant and a growth promotor in the fish as increased intestinal villi height and nutrient absorption were observed ^9^. Moreover, the dietary administration of chitosan nanoparticles can decrease the anaerobic and aerobic bacterial counts in the Nile tilapia intestine and enhance the digestive enzyme activity ^10^. The growth of several freshwater and marine fish species received dietary chitosan, such as rainbow trout (*Oncorhynchus mykiss*), sea bass (*Dicentrarchus labrax*), grey mullet (*Mugil cephalus*), and loach (*Misgurnus anguillicaudatus*)^11^.

Nanotechnology has been approved as a better alternative to other traditional methods in different scientific fields. It is based on the formulation of drugs in biocompatible nanocomposites, including nanocapsules, nanoparticles, and conjugates ^12–19^. Nano-encapsulation protects vitamins against environmental conditions, prolongs their periods of existence, and preserves their properties. Nano vitamins such as VC and VE exhibited higher immune-antioxidant responses compared to free forms, and capsulation with chitosan forming a nanocomposite could improve their activities^20–22^.

This experiment was designed to study the impacts of nanocomposite containing VC and VE on Nile tilapia. Growth parameters, blood analyses, liver enzymes, innate immunity, and serum antioxidant enzymes were recorded and assessed to conclude the efficacy of nanocomposite.

## Materials and methods

### Accommodation and fish feed

Experimental Nile tilapia (*Oreochromis niloticus*) were purchased from private fish farms in Kafrelsheikh Governorate. After tranquilization on the fish farm with 40 mg/L tricaine methanesulfonate (MS-222, Syndel, Canada), fish were transported in clear plastic bags filled with clean and aerated water. Five fish were randomly chosen and examined at the wet laboratory for bacterial and parasitic infections. Pre-acclimatization fish were exposed to an iodine bath of 5% povidone-iodine and produced by the Nile Company for Pharmaceuticals ^23,24^. During the fifteen days of acclimatization, fish were stocked in a fiberglass tank containing dechlorinated clean tap water, and its temperature was 26.5 ± 1.5 °C and pH of 7.4. One-third was changed daily. The basal diet was offered once daily at 0.9:30 a.m., and the feeding rate was 1% of the fish's body weight.

#### Nanoparticles and experimental diets preparation

Nanoparticles were synthesized using the ionotropic gelation method, using 1 g chitosan containing VC 420 mg and/or VE 100 mg ^25^. The characterization was done by TEM high-resolution transmission electron microscopy (JEM1400F HRTEM equipped with a 300 keV beam energy). All ingredients were purchased from the local market: vitamin C (Sciencelab Texas, USA), vitamin E (Glentham Life Science England), and chitosan (Sigma-Aldrich, USA). After soaking in water, A basal fish food was blended, forming a doughy pasta; gelatin (Nutri-B-Gel) produced by Canal Aqua Cure (Port-Said, Egypt) was mixed with feed additives and added to the doughy pasta at a final level of 5% w/w. The diets were left to dry and then cut into similar-sized pellets.

#### Feeding trial

A 420 of the acclimatized Nile tilapia were evenly and randomly stocked into 21 aquaria (20 fish/aquarium), forming four treatments;

1. Control, fish fed a basal diet.
2. Fish-fed dietary bulk vitamin C (VC) at 420 mg/kg fish feed.
3. Fish-fed dietary nanocomposite (VC-NPs) composed of 420 mg VC at a 1 g/kg fish feed dose.
4. Fish were fed dietary bulk vitamin E (VE) at 100 mg/kg fish feed.
5. Fish were fed dietary nanocomposite (VC-NPs) composed of 100 mg of VE at 1 g/kg fish feed.
6. Fish were fed a dietary blend of bulk VC and VE (VCE) at VC 420 mg and VE 100 mg/kg fish feed.
7. Fish were fed dietary nanocomposite (VCE-NPs) composed of 420 mg and VE 100 mg at 1 g/kg fish feed.

Nile tilapia, weighing 31.2 ± 0.36, were fed the trial diets for 8 weeks. Fish were fed at a rate of 5% body weight 6 days per week. The feed amounts were changed at the end of each week according to the achieved body weight. Fish diets were offered twice a day, 09:00 and 03:30 p.m. The chemical composition of the basal diet was moisture 11.1%, crude Protein 42.72%, digestible energy 2955.62 (kcal/kg), ether extracts 5.74%, crude fiber 2.6%, nitrogen-free extract 35.3%, and ash 7.4%. The growth performance of experimental fish was calculated as follows^26^:

$$\text{TWG}=\text{FW}-\text{IW}$$

$$\text{FCR}=\frac{\text{Feed intake}}{\text{TWG}}$$

$$\text{RGR \%}=(\frac{\text{FW}-\text{IW}}{\text{IW}})\times 100$$

where, IW; initial weight, FW; final weight, TWG; Total weight gain, FCR; food conversion rate, RGR; relative growth rate.

### Blood and serum analyses

Blood samples were collected after anesthetizing the fish with tricaine methanesulfonate (MS222; Sigma, St. Louis, MO, USA). Blood was collected from the caudal vein by a syringe moistened with heparin (100 IU/ml). *O. niloticus* red blood cell (RBC) and white blood cell (WBC) counts were determined by a haemocytometer according to Stoskopf^27^, while Hb was calculated by the cyanmethaemoglobin method according to Drubkin^28^.

The concentrations of total protein (TP)^29^ and albumin (Alb)^30^ were measured by colorimetric methods, while globulin concentrations (Glo) were determined by subtracting the albumin concentration from the concentration of total protein albumin.

Liver enzymes: The experimental fish blood was collected from the tail vein and centrifuged to obtain sera, which were used to colorimetrically determine aspartate amino aransaminase (AST) and alanine amino transaminase (ALT) using a spectrophotometer, according to Reitman and Frankel^31^.

### Innate immunity

#### Neutrophils glass-adhesion assay

The oxidative burst activity (OBA) was determined using a nitroblue tetrazolium (NBT) assay, according to Anderson et al.^32^. Briefly, within 15 min. After blood sample collection, one drop of heparinized blood sample was placed onto a cover slip. The coverslips were incubated for 30 min at room temperature (25 °C) in humid chambers to allow the neutrophils to stick to the glass. The coverslips were gently washed with PBS (pH 7.4), and the cells were transferred to a microscope slide containing a 50 μL drop of 0.2% filtered NBT solution (Fluka Buchs, Co. Switzerland). After 30 min of incubation, positive dark-blue stained cells were counted under a light microscope.

#### Phagocytosis assay

To determine the phagocytic activity (PA), firstly, leukocyte isolation was performed according to the method described by Faulmann et al.^33^. Secondly, PA was determined according to Kawahara et al.^34^. *Candida albicans* was prepared from a 24-h-old culture, and the number of *C. albicans* cells was counted to obtain the required concentration of 1 × 106 yeast cells/mL. Separated peripheral leucocytes were adjusted to a 2.5 × 106 viable cells/mL concentration. Then, to each 1 mL of blood leucocytes, 1 mL *C. albicans* suspension was added, and the mixture was incubated in an incubator (CO_2_ 5–10%) at 27 °C/1 h. Smears were prepared and stained with Giemsa stain. A minimum of 100 cells were counted in different fields under the microscope at 1000× magnification.

The PA and the phagocytic index (PI) were calculated using the following equations:

$$\text{PA}=\frac{\text{No}.\text{ of ingesting phagocytes}}{\text{total No}.\text{ of phagocytes}}\times 100$$

$$\text{PI}=\frac{\text{No}.\text{ of ingested }C. albicans \text{cells }}{\text{No}.\text{ of ingesting phagocytes}}$$

#### Serum antibacterial activity

Serum antibacterial activity (SAA) was measured following the procedure of Kajita et al.^35^. Equal volumes (100 μL) of Nile tilapia serum and *Aeromonas hydrophila* bacterial suspension 2 × 10^8^ (CFU) were mixed and incubated for 1 h at 25 °C. A blank control was also prepared by replacing the serum with sterile PBS. The mixture was then diluted with sterile PBS at a ratio of 1:10. The serum-bacteria mixture (100 μL) was plated on blood agar, and the plates were incubated for 24 h at 27 °C. The number of viable bacteria was determined by counting the colonies grown on nutrient agar plates.

### Antioxidants in fish serum

Antioxidants were measured in serum of the experimental Nile tilapia using spectrophotometers (Stat Lab, Germany), malondialdehyde (MDA)^36^, Glutathione peroxidase (GPx)^37^, catalase (CAT)^38^, superoxide dismutase (SOD)^39^, total antioxidant capacity (TAC), oxidized glutathione (GSSG), reduced glutathione (GSH)^40^, glutathione reductase (GR)^41^, and myeloperoxidase (MPO)^42^. The assay kits used for biochemical measurements were purchased from Biodiagnostic and Biotechnology Co., ARE.

### Statistical analyses

The impact of nanocomposites on Nile tilapia's performance was statistically assessed using SPSS software 2022. The mean and standard error of the collected data were determined, and groups were compared using ANOVA and Duncan's Multiple Range with a significant P value of less than 0.05.

### Biosafety

Dead fish were burned using fixed incinerators, following the biosafety measures mentioned by the Pathogen Regulation Directorate (Infectious substances—*A. hydrophila* datasheet)^43^.

### Ethical approval

The above-described methodology was approved by the Ethics Committee at the Animal Health Research Institute and European Union directive 2010/63UE, and all methods were carried out in accordance with relevant guidelines and regulations. This study is reported in accordance with ARRIVE guidelines (https://arriveguidelines.org). This paper does not contain any studies with human participants by any of the authors. No specific permissions were required for access to the artificial pond in wet laboratory Animal Health Research Institute, Kafrelsheikh, Egypt. The field studies did not involve endangered or protected species.

## Results

### Growth performance and feed utilization

In Table 1, Nile tilapia fed on a fortified diet VC and VE achieved high growth performance and feed utilization, using nano-vitamins reinforced with chitosan provided enhancements that had been evaluated in this work. Fish that received nano-fortified feed had higher FW, TWG, DWG, WG%, and RGR with lower FCR and insignificant differences in FI.

**Table 1:** Feed utilization and growth performance.

Items	Control	VC	VC-NPs	VE	VE-NPs	VCE	VCE-NPs
IW (g/fish)	32 ± 1.53	30.3 ± 1.86	31 ± 0.3	32.3 ± 0.3	31.9 ± 0.8	29.9 ± 0.3	30.9 ± 0.24
FW (g/fish)	74.35 ± 2.23^E^	79.2 ± 2.7^D^	85.5 ± 1.2^BC^	82.9 ± 0.5^CD^	89.5 ± 1.6^B^	82.6 ± 0.2^CD^	94.5 ± 0.4^A^
TWG (g/fish)	42.35 ± 0.8^F^	48.8 ± 0.9^E^	54.5 ± 1.13^C^	50.5 ± 0.3^DE^	57.6 ± 1^B^	52.8 ± 0.4^CD^	63.6 ± 0.6^A^
DWG (g/fish)	0.76 ± 0.01^G^	0.87 ± 0.02^F^	0.97 ± 0.02^C^	0.9 ± 0.01^EF^	1.03 ± 0.02^B^	0.94 ± 0.01^DE^	1.13 ± 0.01^A^
RGR%	132.7 ± 4.34^D^	161.8 ± 6.9^C^	175.9 ± 3.8^B^	156.3 ± 1.53^C^	180.8 ± 3.12^B^	176.7 ± 2.98^B^	206.1 ± 3.37^A^
FCR	2.28 ± 0.01^A^	2.02 ± 0.03^B^	1.85 ± 0.13^BC^	1.95 ± 0.07^BC^	1.71 ± 0.07^DE^	1.76 ± 0.09^DE^	1.6 ± 0.06^E^
FI (g)	96.4 ± 1.34	98.7 ± 3.2	101.03 ± 8.2	98.4 ± 4.12	98.6 ± 4.6	92.7 ± 5.4	101.5 ± 3.01
SR %	3.3	6.67	3.3	3.33	6.67	6.67	3.33

Data represented as means ± standard error. Mean values with different letters at the row differ significantly at (P ≤ 0.05). *VC* vitamin C, *VC-NPs* nanocomposite VC, *VE* vitamin E, *VE-NPs* nanocomposite VE, *VCE* vitamin C and E, *VCE-NPs* nanocomposite VCE.

A nanocomposite VCE-NPs had significantly higher TWG (63.6 g), DWG (1.13 g), and RGR (206.1%) with significantly lower FCR (1.6) and insignificant FI (101.5 g) compared to VE-NPs and VCE blend (Table 1).

### Liver enzymes

**Figure 1:**
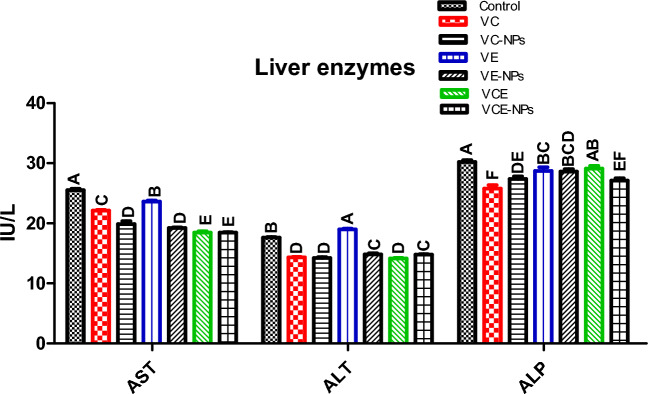
Liver enzymes in *Nile tilapia*. Data represented as means ± standard error. Mean values with different letters at the row differ significantly at (P ≤ 0.05). *VC* vitamin C, *VC-NPs* nanocomposite VC, *VE* vitamin E, *VE-NPs* nanocomposite VE, *VCE* vitamin C and E, *VCE-NPs* nanocomposite VCE.

In Fig. 1, the health of experimental Nile tilapia was assessed by measuring liver enzymes AST, ALT, and ALP. Liver enzymes significantly decreased after 8 weeks of incorporating nanovitamins. Dietary VCE-NPs resulted in the reduction of liver activity that caused significantly lower serum AST and ALT 18.45 IU/L and 14.77 IU/L, respectively, compared to VCE 18.45 IU/L and 14.16 IU/L, respectively (P ≤ 0.05). ALP insignificantly differed between VC-NPs and VE-NPs; VC and VE (Fig. 1).

### Complete blood and serum investigation

In Table 2, blood parameters and serum protein were significantly improved by feeding nanovitamins; RBCs were 4.2 × 106/μL, 3.8 × 106/μL, and 3.55 × 106/μL in fish groups supplemented with VCE-NPs, VC-NPs, and VE-NPs, respectively. The levels of Hb and PCV showed the same trend for RBCs g/dL%. Feeding dietary VCE-NPs, VE-NPs, and VC-NPs resulted in significantly higher WBCs, 46.15, 42.9, and 44 × 103/μL, respectively. TP was significantly higher at 6.38 g/dL by VCE-NPs supplementation, followed by VC-NPs at 6.38 g/dL, whereas no significance was recorded between the other groups. A significant rise in Glo level was recorded in fish serum that received dietary VCE-NPs and VE-NPs followed by VC-NPs. In contrast, Alb had insignificant differences in serum in the experimental groups.

**Table 2:** Blood analyses of the experimental Nile tilapia.

Items	Control	VC	VC-NPs	VE	VE-NPs	VCE	VCE-NPs
RBCs 10^6^/μL	3 ± 0.05^E^	3.15 ± 0.03^D^	3.8 ± 0.04^B^	2.99 ± 0.02^E^	3.55 ± 0.03^C^	2.98 ± 0.04^E^	4.2 ± 0.06^A^
Hbg/dL	10.55 ± 0.15^E^	11.5 ± 0.1^D^	14.6 ± 0.15^B^	12.3 ± 0.06^C^	14.6 ± 0.1^B^	11.5 ± 0.12^D^	15.45 ± 0.22^A^
PCV%	29.9 ± 0.5^E^	33.13 ± 0.36^D^	39.2 ± 0.5^B^	35.9 ± 0.22^C^	40.13 ± 0.23^B^	34.5 ± 0.4^CD^	43.6 ± 0.8^A^
WBCs10^3^/μL	32.8 ± 0.18^G^	33.8 ± 0.14^E^	42.9 ± 0.2^C^	33.37 ± 0.33^FG^	44 ± 0.12^B^	34.7 ± 0.2^D^	46.15 ± 0.2^A^
TPg/dL	5.15 ± 0.08^C^	5.34 ± 0.03^C^	5.8 ± 0.04^B^	5.4 ± 0.01^C^	6.52 ± 0.07^C^	5.23 ± 0.14^C^	6.38 ± 0.1^A^
Albg/dL	3.18 ± 0.04	3.28 ± 0.08	3.3 ± 0.01	3.19 ± 0.09	3.33 ± 0.05	3.35 ± 0.01	3.32 ± 0.06
Glog/dL	1.97 ± 0.11^C^	2.06 ± 0.06^C^	2.5 ± 0.03^B^	2.2 ± 0.08^C^	3.19 ± 0.09^A^	2 ± 0.16^C^	3.06 ± 0.06^A^

Data represented as means ± standard error. Mean values with different letters at the row differ significantly at (P ≤ 0.05). *VC* vitamin C, *VC-NPs* nanocomposite VC, *VE* vitamin E, *VE-NPs* nanocomposite VE, *VCE* vitamin C and E, *VCE-NPs* nanocomposite VCE.

### Innate immunity

**Figure 2:**
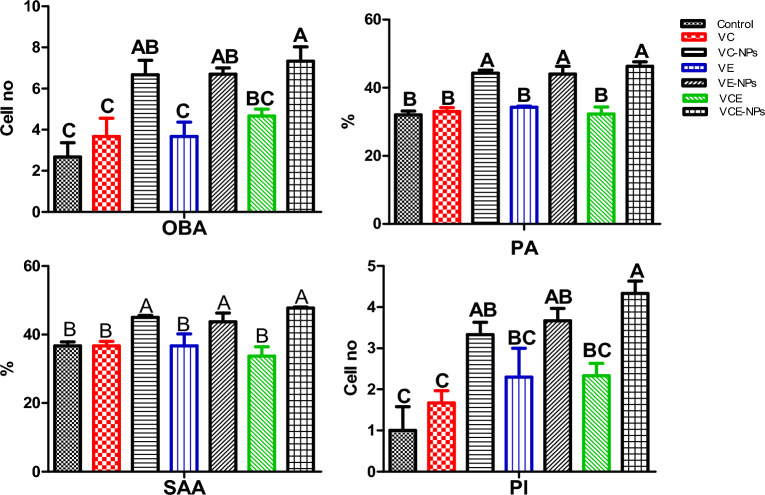
Investigation of innate immunity. Data represented as means ± standard error. Mean values with different letters at the row differ significantly at (P ≤ 0.05). *VC* vitamin C, *VC-NPs* nanocomposite VC, *VE* vitamin E, *VE-NPs* nanocomposite VE, *VCE* vitamin C and E, *VCE-NPs* nanocomposite VCE.

In Fig. 2, the activity of immune cells (heterophils) was assessed by measuring OBA which revealed that dietary VCE-NPs, VE-NPs, and VC-NPs (7.33, 6.7, and 6.4.67 Cell.no.) were significantly higher than vitamins VCE, VE, and VC (7.33, 3.67, and 3.67 Cell.no.), respectively, compared to the control fish 2.67 Cell.no. (P ≤ 0.05). The activity of phagocytic cells (PA and PI) and serum antibacterial (SAA) showed the same trend as that of OBA.

### Activity of antioxidants in fish serum

**Figure 3:**
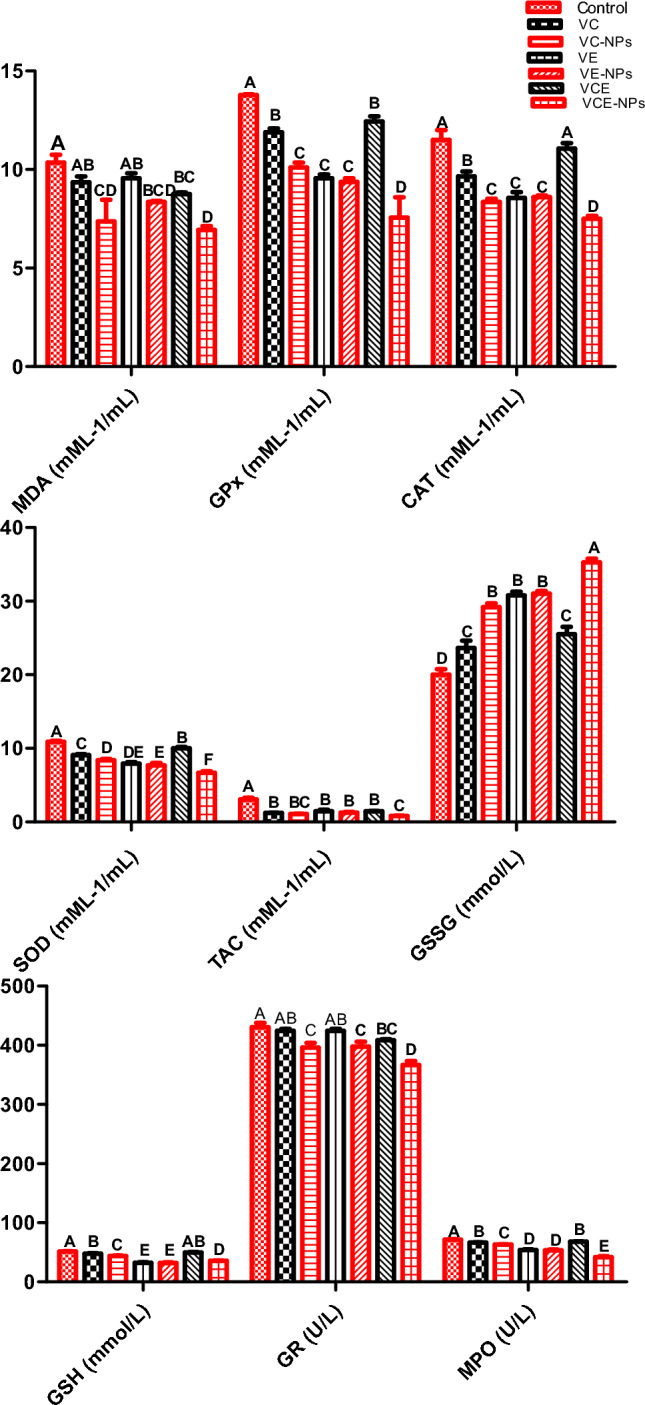
Serum antioxidant biomarkers of the experimental Nile tilapia. Note: Data represented as means ± standard error. Mean values with different letters at the row differ significantly at (P ≤ 0.05). *VC* vitamin C, *VC-NPs* nanocomposite VC, *VE* vitamin E, *VE-NPs* nanocomposite VE, *VCE* vitamin C and E, *VCE-NPs* nanocomposite VCE, *MDA* Malondialdehyde, *GPX* Glutathione peroxidase, *CAT* Catalase, *SOD* Superoxide dismutase, *TAC* Total antioxidant capacity, *GSSG* oxidized glutathione, *GSH* reduced glutathione, *GR* glutathione reductase, *MPO* Myeloperoxidase activity.

In Fig. 3, the level of MDA, GPx, CAT, SOD, TAC, GSSG, GSH, GR, and MPO in fish serum were measured to assess the efficacy of feed additives. Both forms of VE had lesser impacts on antioxidant enzymes compared to both forms of VC and VCE. MDA was significantly decreased with VC-NPs and VCE-NPs to 7.37 and 6.93 mML-1/mL, respectively. GPx was significantly decreased with VCE-NPs, followed by VC-NPs and VCE to 7.55, 10.1, and 9.37 mML-1/mL, respectively. CAT was significantly decreased with VCE-NPs and VC-NPs, followed by VE-NPs to 7.5, 8.36, and 8.6 mML-1/mL, respectively. SOD was significantly decreased with VCE-NPs 6.68 mML-1/mL, respectively. GSH and MPO had the same pattern of SOD. TAC was significantly decreased with VCE-NPs 0.82 mML-1/mL, followed by the other groups, which were significantly higher than the control. GSSG was significantly increased with VCE-NPs followed by VE-NPs and VC-NPs, 35.7, 31, and 29.17 mML-1/mL, respectively. GR had the same pattern as GSSG.

## Discussion

Vitamins C and E are micronutrients required for optimal fish growth and feeding utilization; VC promotes the performance of animals fed a diet lacking VE and vice versa, as fish could avoid growth retardation caused by a diet lacking VC by 0.1 g/kg of VE ^4,44,45^.

Experimental Nile tilapia received dietary VC and VE at a dose of 420 and 100 mg/kg fish feed, respectively, achieving high growth performance and feed utilization compared to the control; meanwhile, VCE had superiority. Accordingly, dietary VC at a level of 300 mg/kg diet significantly boosted the performance and feed consumption of Nile tilapia ^46^, rainbow trout under crowding conditions ^47^, WG and SGR of zebrafish (*Danio rerio*), juvenile Korean rockfish (*Sebastes schlegeli*), rohu fry (*Labeo rohita*), and soft-shelled turtle (*Pelodiscus sinensisfed*)^48,49^. In conformity, Zanella et al.^50^ observed that pacu fish which was exposed to 72 h of food limitation and received a dietary VC (200 μM, 24 h) the diameter of muscle cells increased, and the expression of genes related to anabolic and cell proliferation was significantly improved. Similarly, a combination of VC and VE could maximize the growth (WG) and feed utilization (SGR) of hybrid male abalone (*Haliotis fulgens ♂* × *H. discus hannai ♀*)^51^.

Moreover, chitosan's effectiveness was better as an oral delivery vehicle of selenium, folic acid, and lemon essential oil in fish ^52,53^. Experimental Nile tilapia received dietary VC-NPs and VE-NPs reinforced and encapsulated with chitosan showed an enhanced FW, TWG, DWG, WG%, and RGR with lower FCR and insignificant differences in FI compared to the control fish and those supplemented with dietary VC, and VE. The Nile tilapia-fed dietary composite (VCE-NPs) achieved higher growth parameters and feed utilization than all treatments. Similarly, WG and SGR were significantly increased in shrimp-fed dietary VC-loaded chitosan nanoparticles (VC-NPs), along with a high survival rate^54^. Nano-sized vitamins VC and VE have higher bioavailability due to high molecular level dispersion, boosting the growth of rainbow trout^55^ and Nile tilapia^45^. Also, dietary chitosan nanoparticles enhanced growth performance by protecting VC and VE from the intestinal tract's adverse environment, increasing nanoparticles' existence period, increasing the possibility of absorption via intestinal epithelium compared to micro-chitosan and free vitamins, and inducing the activities of digestive enzymes and inhibit pathogenic bacteria in the Nile tilapia ^56^, Silver carp (*Hypophthalmichthys molitrix*) ^57^, common carp (*Cyprinus carpio*)^58^, hybrid tilapia (*Oreochromis niloticus* ♀ × *Oreochromis aureus* ♂) ^59^.

The fish liver's malfunction, damage, and injuries could be observed by reporting serum AST and ALT ^60^. The level alterations of serum ALP could be attributed to immunity and immune defense systems ^61^, and abnormality occurred in the signal membrane transport system^62^. In this work, AST, ALT, and ALP were significantly decreased in Nile tilapia serum, which received dietary nanovitamins VCE-NPs for eight weeks. Similarly, Ahmed et al.^20^ conducted a 70-day feeding trial of Nile tilapia with an average weight of 14.74 ± 0.06 g fed chitosan VE nanocomposite (300 mg/kg), the serum AST and ALT levels were decreased. Also, the ALT and AST levels were decreased in Nile tilapia serum after supplementation with a 5 g chitosan/kg diet ^56^. Both vitamins VC and VE are crucial antioxidants that can scavenge ROS released under stress conditions and minimize damage to the tissue of fish liver due to oxidative injury ^5,8^.

Blood parameters and serum TP were expected to significantly improve in experimental Nile tilapia after the growth and feed utilization improvements, mainly with those received composite of chitosan nanocomposite VC-NPs, VE-NPs, and VCE-NPs. Other authors made similar observations on different fish species, such as rainbow trout ^11^. Interestingly, VE could stimulate erythropoiesis by increasing the Hb content of RBC^63^. In addition, antioxidants such as VC and VE inhibit the oxidative damage of cell membrane polyunsaturated fatty acids protecting the fish cells^8,63^. Conversely, Naderi et al.^64^ recorded a decline in the levels of PCV and Hb in rainbow trout serum fed a dietary VE and Nano-Se at doses of 1 mg/kg and 500 mg/kg, respectively. Such disagreements might be due to differences in the nature and amount of supplements, feeding period, diet ingredients, or synergistic impact of supplements that could act differently from their free form. Accordingly, feeding a diet with high VC and VE concentrations for 40 days could significantly increase the TP levels in Nile tilapia serum^65^. In accordance, it was reported a considerable rise of the TP and GLO values in the serum of Nile tilapia supplemented with dietary chitosan nanoparticles at a concentration of 5 g/kg diet ^56,66^.

Phagocytosis, including PA and PI, is a defensive response of fish against foreign and infectious agents^67^. In this study, the innate immune was improved in experimental Nile tilapia that received dietary VC-NPs, VCE-NPs, and VE-NPs manifested by increased OBA, phagocytic cells (PA and PI), and serum antibacterial (SAA), along with significant high WBCs were 46.15, 42.9, and 44 × 103/μl, respectively. Accordingly, OBA is produced by the phagocytic cells and is associated with killing the bacterial pathogen. OBA of blood neutrophils defends against pathogenic organisms ^68,69^. Increased SAA activity in fish correlated with immune cell count, which produces lysozyme ^70^. Similarly, shrimp fed a composite chitosan VC-NPs showed a considerable improvement in immunological responses such as OBA, WBCs, and disease resistance ^54^. Also, Nile tilapia received dietary VC, and their phagocyte cells increased in number, which caused an upsurge in lysozyme production, showing a high bactericidal effect ^46^. Dietary VE could boost resistance against several pathogens by stimulating PA, PI, and SAA and inducing antibody production ^71^. In addition, chitosan could modulate the immune responses by stimulating macrophage activity release of cytokine and antibody responses**,** including enzymes, blood proteins, and antibodies) ^72,73^. It could also penetrate bacterial cell walls, releasing cytoplasmic contents^74^.

Serum of experimental Nile tilapia showed significantly low values of MDA, GPX, CAT, SOD, TAC, GSSG, GSH, GR, and MPO, mainly in those who received dietary VC-NPs, VE-NPs, and VCE-NPs. The decline of antioxidant enzymes may be due to the properties of VE that could inhibit lipid oxidation through scavenging ROS ^75^. Similarly, MDA has significantly declined in the liver and blood of Nile tilapia reared under the stress of high stocking density and fed dietary VC-NPs and VE ^20^. Accordingly, VE can scavenge the reactive oxygen species released in stress conditions and minimize liver damage^76^. Similarly, Asaikkutti et al.^54^ reported that nanocomposite of chitosan VC is an important antioxidant, LPO and GPx activity showed a significant increase in non-challenged shrimps, and the challenged control shrimp had the lowest activity. In accordance, Yilmaz et al.^77^ found that natural products (plant-origin) could induce gene expression of antioxidant-related genes (SOD, CAT, and GPx) in the liver scavenging the generated ROS.

On the contrary, antioxidant activity in the kidney and liver tissues of rohu fish was higher in those fed with the chitosan NPs at a 1 g/kg diet ^78^. However, those were challenged against *A. hydrophila* infection, which consumes more antioxidants. These differences may be attributed to sampling time, infection challenge, and experimental design.

## Conclusion

The nanocomposite VC, and VE could be incorporated into the *Nile tilapia* diet without any health hazards. Dietary nanocomposites enhanced Nile tilapia's growth with better feed utilization, where FCR was 1.6 in the VCE-NPs group. They could modulate the activity of phagocytic cells and antibacterial serum of fish that received dietary VCE-NPs, VE-NPs, and VC-NPs were significantly higher than bulk-vitamins VCE, VE, and VC.as were increased in fish. The serum levels of antioxidant biomarkers indicated that nanocomposite could protect Nile tilapia against the generated ROS.

## Data Availability

Data is available on request from the corresponding author.
